# A SHAP-interpretable machine learning framework for predicting delayed discharge in ambulatory total knee arthroplasty: comparative validation of 14 models

**DOI:** 10.3389/fmed.2025.1714792

**Published:** 2025-11-05

**Authors:** Jiabin Feng, Fei Yuan, Pan Luo, Rang Chen, Binghu Jiang, Bin Li, Fang Luo, Li Sun, Bo Li

**Affiliations:** ^1^Department of Orthopaedic Surgery, Guizhou Provincial People’s Hospital, Guiyang, Guizhou, China; ^2^College of Orthopedics and Traumatology, Guizhou University of Traditional Chinese Medicine, Guiyang, Guizhou, China

**Keywords:** machine learning, SHAP, delayed discharge, ambulatory total knee arthroplasty, risk prediction, interpretable artificial intelligence, perioperative management

## Abstract

**Background:**

The rising global demand for total knee arthroplasty (TKA) has accelerated the shift toward ambulatory surgery, aimed at same-day or next-day discharge. However, significant variability in discharge protocols and high rates of delayed discharge in unselected patients challenge its widespread implementation. This study develops an interpretable machine learning framework to preemptively identify risk factors for delayed discharge in ambulatory TKA.

**Methods:**

This retrospective study analyzed data from 449 patients who underwent ambulatory total knee arthroplasty between September 2021 and June 2024. Fourteen machine learning models were developed and validated using preoperative variables selected via LASSO and multivariate regression. The dataset was split into training (70%) and validation (30%) sets, with hyperparameter tuning performed through grid search and 5-fold cross-validation. SHAP analysis was applied to interpret feature importance in the optimal model.

**Results:**

Analysis of 449 patients identified five key predictors—ejection fraction, preoperative eGFR, preoperative ESR, diabetes mellitus, and Barthel Index—via LASSO and multivariate regression. Among 14 machine learning models, CATBoost exhibited optimal performance, with an AUC of 0.959 in training and 0.832 in validation, supported by high net benefit in decision curve analysis. SHAP analysis identified EF and preoperative ESR as the most influential features, confirmed risk directionality for low EF and low Barthel Index, and revealed nuanced interactions, such as the inverse relationship of EF with risk, enhancing model interpretability.

**Conclusion:**

This study establishes that machine learning, particularly the CATBoost model, effectively predicts delayed discharge in ambulatory total knee arthroplasty using five key preoperative variables. SHAP analysis further enhanced model interpretability by revealing feature interactions, such as the modulating role of ejection fraction. These predictors enable improved risk stratification and personalized discharge planning, supporting optimized resource use and patient management. While limitations like single-center data require cautious interpretation, the findings highlight the potential of predictive analytics for clinical deployment. Further validation in diverse settings is warranted to translate these findings into clinical practice.

## 1 Introduction

Accelerated demographic aging has precipitated a marked increase in knee osteoarthritis (KOA), a progressive degenerative joint disease now affecting over 17% of adults aged ≥ 40 years (1). This condition, manifesting through chronic pain and functional decline, ranks among the primary disability drivers in elderly cohorts. Total knee arthroplasty (TKA) stands as the definitive surgical solution for end-stage KOA, restoring mobility globally. Projected demand surges—exemplified by 3.48 million annual procedures anticipated in the US by 2030 (1)—are amplified by aging populations and rising obesity. Contemporary research prioritizes three domains: surgical precision, anesthesia protocols, and postoperative management, collectively targeting shortened hospitalization with improved outcomes (2–4). Economic pressures further propel TKA’s migration to ambulatory centers for cost-effective high-volume delivery (2).

Ambulatory (day-case) surgery entails admission-to-discharge within one calendar day. The International Association for Ambulatory Surgery (2003) mandates ≤ 24-h hospitalization excluding outpatient procedures (3). China’s protocol (2015) requires pre-admission assessments, permitting ≤ 48-h stays for medical necessity (4).

No universal standard governs ambulatory TKA, with discharge timelines ranging from same-day (5–7) to 48-h (8, 9) periods, and some studies lacking formal criteria (10, 11). The US system exhibits reimbursement dichotomies: insurance classification dictates surgical categorization, wherein misclassification incurs penalties—prompting private insurers to adopt 48-h benchmarks (12). China’s extended recovery practices align with this 48-h threshold.

Ambulatory TKA demonstrates non-inferior safety and superior patient satisfaction versus inpatient models (13–15), optimizing resource allocation via accelerated recovery, cost reduction, and enhanced bed turnover. While high-volume centers report 80–100% same-day discharge in curated cohorts (excluding comorbidities, elderly, and high-BMI patients) (16–18), real-world data reveals constrained applicability: a prospective multicenter study documented mere 15% same-day discharge among 557 unselected candidates (19). This disparity necessitates identifying modifiable risk factors for discharge delays.

## 2 Materials and methods

### 2.1 Data source, patient selection, and ethical considerations

This study retrospectively collected and analyzed medical records of all patients undergoing ambulatory Total knee arthroplasty at Guizhou Provincial People’s Hospital between September 2021 and June 2024. All surgical procedures were performed by the same operating surgeon. Patients were included if they met the following criteria: 1. availability of complete medical records required for the study, and 2. having undergone same-day unilateral total knee arthroplasty. Patients were excluded if they had undergone simultaneous bilateral total knee arthroplasty during the same operative session.

**FIGURE 1:**
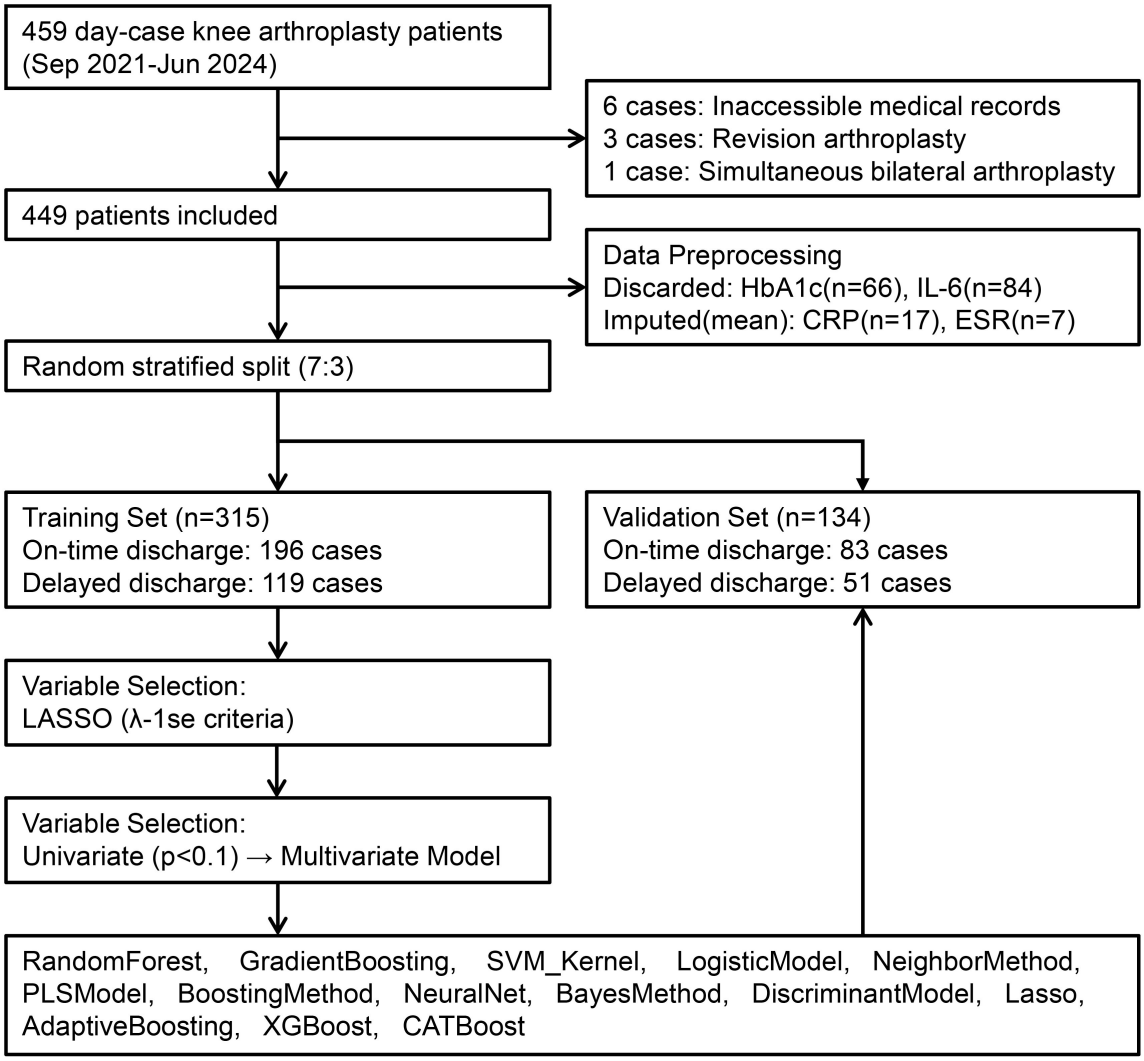
Patient enrollment flowchart and cohort construction.

Based on these criteria, 449 patients were identified for model development, categorized by hospitalization time into an on-time discharge group (≤ 48 h) and a delayed discharge group (> 48 h). This study was reviewed and approved by the Ethics Committee of [Guizhou Provincial People’s Hospital] (approval number: Lun-Shen-KeYan-2024-186), in accordance with the Declaration of Helsinki. All participants provided informed consent. As this was a retrospective analysis, it was not registered as a clinical trial. Patient data underwent compliant de-identification procedures to ensure privacy protection, with only anonymized information used. A flowchart illustrating the enrollment of the study population is shown in Figure 1.

### 2.2 Input variables and data processing

As predictors, data were collected from the electronic medical record system of Guizhou Provincial People’s Hospital for all cases meeting the predefined inclusion and exclusion criteria, utilizing standardized forms to record variables categorized into six domains: sociodemographics (Gender, Age, Occupation, BMI, Educational Attainment), admission metrics (Body Temperature, Pulse Rate, Systolic Blood Pressure, Diastolic Blood Pressure, Barthel Index), medical history (Smoking Status, Alcohol Use, Hypertension, Diabetes Mellitus, Osteoporosis, Emphysema, pneumonia, History of Cerebral Infarction, Coronary Artery Disease), preoperative tests [Ejection Fractions (EF), Preop-Hb, Preop-PLT, Preop-CRP, Preop-eGFR, Preop-Alb, Preop-ESR], knee-specific factors (Disease Duration, Knee Flexion Angle, Knee Extension Deficit, Contralateral Arthroplasty History, Knee Deformity, Resting VAS Score, Mobility Aid Requirement), and surgical parameters [American Society of Anesthesiologists (ASA), Surgical Laterality, Anesthesia Type]; for missing value handling, cases were excluded if the outcome variable (length of stay, LOS) was missing or if multiple covariates exhibited simultaneous missingness, while variables missing completely at random with low missingness rates were imputed using mean values for continuous data or mode for categorical data, and those with high missingness rates were discarded entirely.

### 2.3 Quality control

To maintain processing integrity, a staged workflow segregated data entry, de-identification, and analysis among independent researchers, implementing standardized protocols where: dual-track recording was executed by competency-certified personnel using structured electronic forms; comprehensive cross-validation minimized entry discrepancies; a 20% random subset underwent auditing for accuracy verification; and anonymized datasets were processed through chain-of-custody protocols for blinded statistical analysis.

### 2.4 Statistical analysis

All data analyses were conducted in R version 4.4.3 (20) using the compareGroups package. Continuous variables with normal distribution were expressed as mean ± standard deviation (SD), while non-normally distributed variables were summarized as median with interquartile range [Median (Q1, Q3)]. Categorical variables were presented as counts and percentages [n (%)]. Between-group comparisons employed: independent *t*-tests for normally distributed continuous data; Wilcoxon Rank-Sum tests for non-normally distributed continuous variables; chi-square tests for unordered categorical data; and Mann-Whitney U tests for ordered categorical variables. A uniform significance threshold of α = 0.05 was applied for all inferential tests.

### 2.5 Model development and validation

The variable selection process was conducted through a sequential, two-step approach to ensure both robustness and interpretability. First, least absolute shrinkage and selection operator (LASSO) regression with the 1SE lambda criterion was applied to preliminarily screen variables, enhancing model sparsity and reducing overfitting. Second, variables retained from the LASSO screening were further analyzed using univariate logistic regression, with those significant at *p* < 0.1 included in a subsequent multivariate logistic regression model. A relaxed alpha level (*p* < 0.1) was intentionally chosen for the univariate and multivariate regression stages to adopt a more conservative approach to feature selection, thereby reducing the risk of excluding potentially relevant predictors prior to machine learning modeling. Finally, variables that remained statistically significant (*p* < 0.1) in the multivariate model were selected as the final set of predictors for all subsequent model construction.

Fourteen machine learning algorithms—RandomForest, GradientBoosting, SVM_Kernel, LogisticModel, NeighborMethod, PLSModel, BoostingMethod, NeuralNet, BayesMethod, DiscriminantModel, Lasso, AdaptiveBoosting, XGBoost, and CATBoost—were subsequently implemented. The dataset was partitioned into training (70%) and independent validation (30%) subsets.

Hyperparameter optimization was performed using grid search with 5-fold repeated cross-validation. Model performance was evaluated based on metrics derived from confusion matrices (sensitivity, specificity, accuracy, PPV, NPV, F1-score, Youden’s index), area under the ROC curve (AUC), and residual analysis. Visualization included performance metric tables, line plots, ROC curves, forest plots for AUC comparisons, and residual plots.

### 2.6 Feature importance

SHapley Additive exPlanation (SHAP) analysis quantified predictor contributions using game-theoretic principles (21), resolving AI’s “black-box” limitation through individualized feature-deviation decomposition; for the optimal model, interpretability was enhanced by generating three specialized visualizations: feature dependence plots examining non-linear predictor-outcome relationships, SHAP beeswarm plots revealing individual prediction distributions clustered by feature impact, and ranked feature importance barplots prioritizing clinically dominant variables.

## 3 Results

### 3.1 Subject selection and data processing

Ten patients were excluded per predefined criteria, resulting in 449 ambulatory knee arthroplasty candidates randomly allocated to training (*n* = 315) and validation (*n* = 134) sets at 7:3 ratio (Figure 1). Variables with critical missingness [glycated hemoglobin (HbA1c): *n* = 66; interleukin-6: *n* = 84] were discarded, while continuous covariates (preoperative CRP: *n* = 17; ESR: *n* = 7) underwent mean imputation.

### 3.2 Baseline characteristics

Supplementary Table 1 demonstrated that significant differences (*p* < 0.05) were observed between the on-time discharge (≤ 48 h) and delayed discharge (> 48 h) cohorts in terms of EF, knee flexion angle, knee extension deficit, preoperative CRP, preoperative eGFR, preoperative ESR, pulse rate, diabetes mellitus, and Barthel Index.

A comparative analysis of the training and validation sets revealed that, except for diastolic blood pressure, which exhibited a significant difference between the two sets (*p* = 0.034), no significant differences were observed for the remaining covariates and outcome stratifications (*p* > 0.05), confirming the successful randomization and reliability of the model.

### 3.3 Strategy and initial screening via lasso regression

**FIGURE 2:**
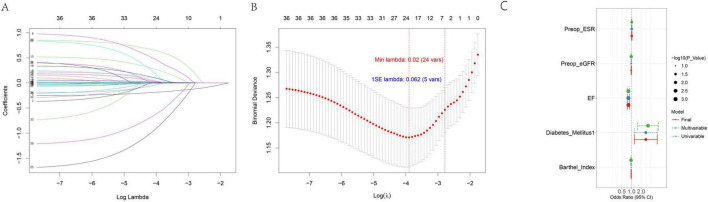
Variable selection via Lasso regression **(A,B)** and final predictor forest plot **(C)**.

To select optimal predictors for subsequent machine learning model construction, a two-stage strategy combining Lasso regression and stepwise univariate-multivariate logistic regression was adopted. Lasso regression was first applied to filter candidate variables, leveraging penalization to mitigate multicollinearity and overfitting. Results are presented in Figures 2A,B: Figure 2A (coefficient path plot) tracks coefficient changes as the regularization parameter (λ) decreases (variables with coefficients shrunk to zero were excluded), while Figure 2B (10-fold cross-validation [CV] curve) marks λ_min_ (minimum CV error) and λ_1se_ (within 1 standard error of λ_min_). For model parsimony and robustness, λ_1se_ was selected as the optimal λ, retaining 5 variables with non-zero coefficients for further selection.

### 3.4 Further selection via stepwise logistic regression

The 5 Lasso-retained variables underwent univariate logistic regression; those with *P* < 0.1 (to avoid missing potential predictors) were included in stepwise multivariate logistic regression (using the same *P* < 0.1 threshold for forward selection). All 5 variables passed both selection steps, confirming their independent predictive relevance. Detailed results—including the odds ratio (OR) with 95% confidence interval (95% CI) and *P*-value—are summarized in Supplementary Table 2, with associations visualized in Figure 2C (forest plot). These findings confirm the 5 variables—EF, preoperative eGFR, preoperative ESR, diabetes mellitus, and Barthel Index—are suitable for inclusion in subsequent machine learning model construction.

### 3.5 Comparative model performance and CATBoost superiority

Hyperparameter optimization via grid search significantly enhanced model performance across all algorithms. The optimal hyperparameter combinations for each model, determined through five-fold cross-validation based on AUC, are summarized in Supplementary Table 3.

**FIGURE 3:**
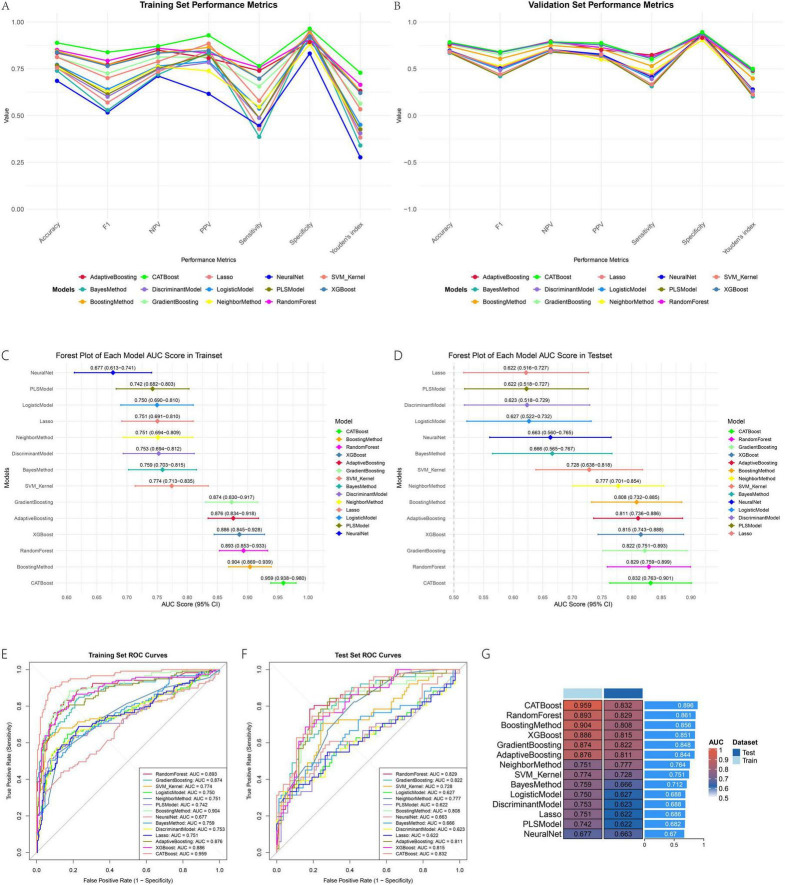
Model performance metrics **(A,B,E,F)**, AUC comparisons **(C,D)**, and summary heatmap **(G)**.

All evaluated machine learning models demonstrated robust predictive performance for the target outcome. Among them, tree-based ensemble methods—particularly CATBoost—exhibited superior discriminative ability and generalizability. In the training set, CATBoost achieved the highest AUC (0.959; 95% CI: 0.938–0.980), followed by BoostingMethod (AUC = 0.904) and RandomForest (AUC = 0.893). The forest plots illustrating AUC values across training and validation sets (Figures 3C,D) visually underscore the consistency of these results. This performance was well maintained in the validation set, where CATBoost also led with an AUC of 0.832, indicating excellent generalization with minimal overfitting. The ROC curves (Figures 3E,F) further confirmed its strong classification capability, with curves closest to the top-left corner in both datasets. Traditional methods such as Logistic Regression (AUCtest = 0.627) and PLS (AUCtest = 0.622) underperformed relative to ensemble models. The AUC heatmap (Figure 3G) visually emphasized the consistent advantage of tree-based algorithms, with CATBoost showing the highest average AUC. Beyond discrimination, CATBoost also exhibited well-balanced sensitivity (0.608) and specificity (0.892), along with the highest Youden’s index (0.499), supporting its clinical utility.

**FIGURE 4:**
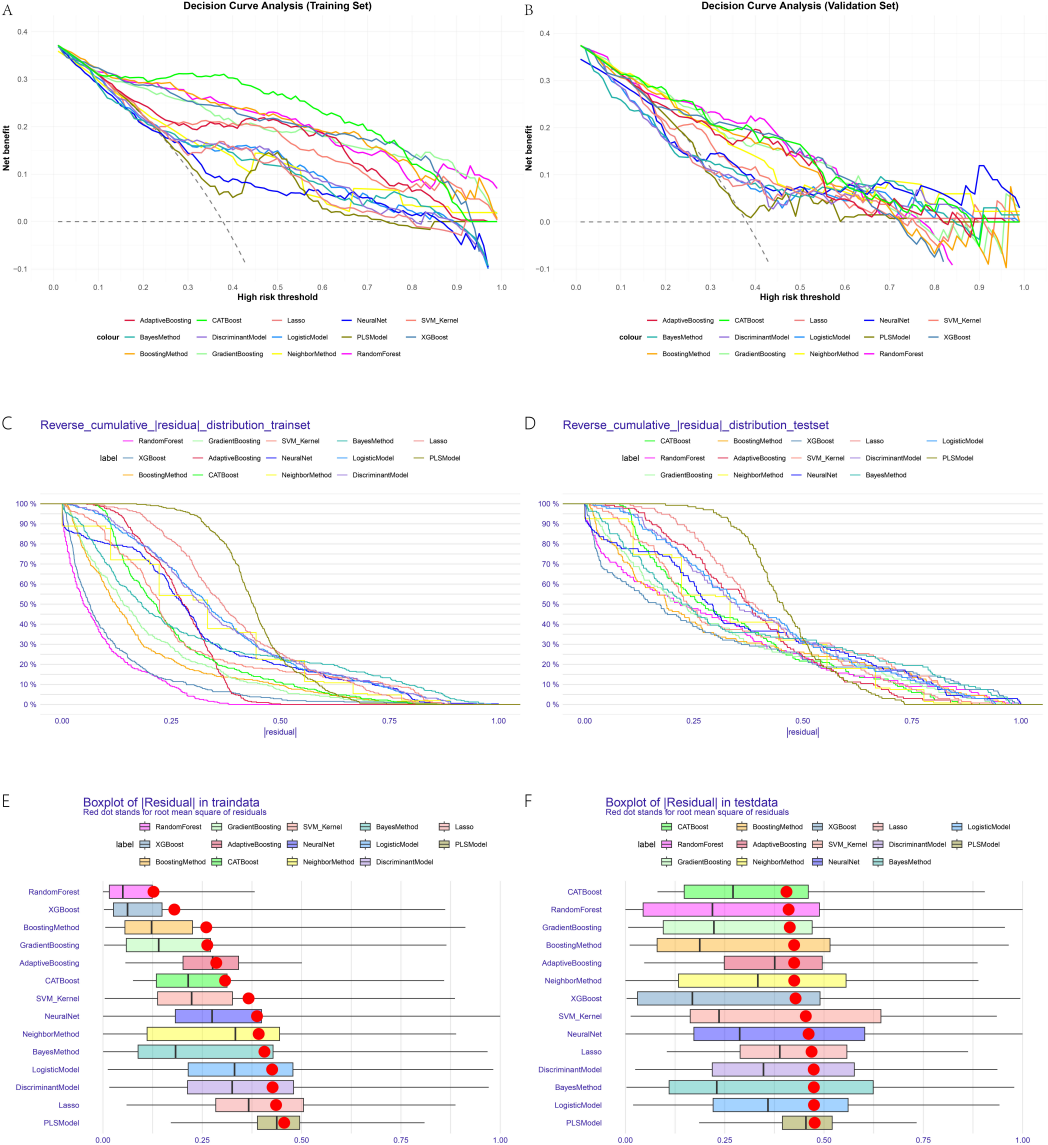
Decision curve analysis **(A,B)** and residual analysis **(C–F)** across all models.

The line plots for sensitivity, specificity, and Youden’s index in training and validation sets (Figures 3A,B) provide a dynamic view of these metrics across thresholds. Decision curve analysis (Figures 4A,B) demonstrated that CATBoost provided superior clinical utility across a wide range of risk thresholds, yielding higher net benefits compared to other models in both training and validation sets. Residual analysis revealed that CATBoost maintained intermediate performance in prediction consistency. The inverse residual cumulative distribution Plot (Figures 4C,D) and residual box plot (Figures 4E,F) showed that CATBoost achieved reasonable prediction error distribution, neither the best nor the worst among all models, but with acceptable error characteristics for clinical application. Overall, CATBoost demonstrated the best combination of discriminative performance, clinical utility, and generalization capability, making it the optimal model for this prediction task despite its intermediate performance in residual analysis.

### 3.6 Detailed evaluation and clinical applicability of CATBoost

**FIGURE 5:**
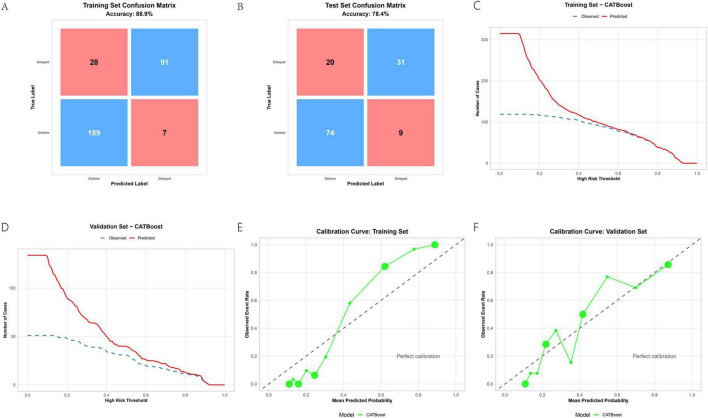
CATBoost model evaluation: confusion matrices **(A,B)**, decision curves **(C,D)**, and calibration plots **(E,F)**.

Building upon its overall superiority, the optimal model, CATBoost, demonstrated robust performance across multiple evaluation metrics in both training and validation sets. The confusion matrices (Figures 5A,B) revealed strong predictive accuracy, with CATBoost achieving 88.9% accuracy in the training set and 78.4% in the validation set. These results indicate a well-generalized model with minimal overfitting. Decision curve analysis (Figures 5C,D) further affirmed the clinical utility of CATBoost, showing substantially higher net benefit across a wide range of risk thresholds compared to alternative strategies (“treat all” or “treat none”) and other models. This suggests that predictions from CATBoost are clinically actionable and can effectively support decision-making in practical settings. Calibration curves (Figures 5E,F) indicated that the predicted probabilities by CATBoost aligned well with observed outcomes, particularly in the training set. While some modest miscalibration was observed in the validation set—reflecting the common challenge of maintaining perfect probability alignment in external data—the overall performance remained clinically acceptable. Together, these results reinforce that CATBoost not only achieved high predictive performance (AUCtrain = 0.959, AUCtest = 0.832), but also demonstrated robust clinical utility and reliability, supporting its use as a promising tool for predicting delayed discharge in ambulatory TKA in clinical contexts.

### 3.7 SHAP analysis reveals model interpretability and key feature relationships

**FIGURE 6:**
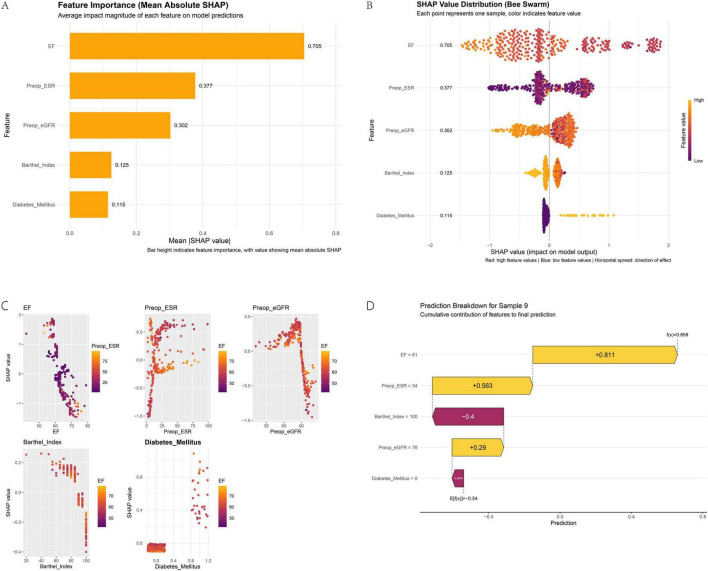
SHAP analysis for model interpretability: feature importance **(A)**, dependence plots **(B)**, interaction effects **(C)**, and a local explanation **(D)**.

Based on the SHAP analysis in Figure 6, key interpretability insights are elucidated. Figure 6A identifies EF and Preop-ESR as the most influential features. Figure 6B confirms risk directionality, demonstrating that progressively lower EF values and lower Barthel Index scores are associated with higher risk (positive SHAP values). Figure 6C reveals nuanced feature interactions: EF exhibits an inverse relationship with delayed discharge risk, where steadily higher EF values are generally associated with reduced risk; however, when Preop-ESR is high, an increase in EF may slightly elevate risk probability, though the interaction effect is minimal. Conversely, elevated Preop-ESR coupled with higher EF significantly decreases overall risk. The interaction between Preop-eGFR and EF is negligible. Barthel Index is inversely associated with risk, with values exceeding 90 conferring a protective effect, and its interaction with EF on risk probability is limited. The presence of Diabetes mellitus substantially increases risk, and this effect is amplified at higher EF values. Figure 6D provides a local explanation for sample #9, illustrating how each feature shifts the prediction from the base value. Absence of Diabetes Mellitus (−0.0658) and Barthel Index = 100 (−0.4) reduce risk, while EF = 61 (+ 0.811), Preop-ESR = 34 (+ 0.563), and Preop-eGFR = 76 (+ 0.29) increase it, resulting in a final prediction of 0.658 (exceeding the 0.50 threshold).

## 4 Discussion

### 4.1 Model development and validation with CATBoost superiority

We developed and validated multiple machine learning models to predict delayed discharge in ambulatory TKA patients, employing a rigorous variable selection process via stepwise logistic regression. This approach confirmed the independent predictive relevance of five key variables—EF, preoperative eGFR, preoperative ESR, diabetes mellitus, and Barthel Index—which were subsequently used in model construction. Hyperparameter optimization via grid search enhanced performance across all algorithms, with tree-based ensemble methods, particularly CATBoost, demonstrating superior discriminative ability and generalizability. CATBoost achieved the highest AUC in both training (AUC = 0.959) and validation sets (AUC = 0.832), indicating robust generalization compared to alternatives like Logistic Regression (AUC = 0.627), which exhibited substantial underfitting.

This performance can be contextualized against existing prediction tools for similar outcomes. For instance, a study by Turcotte et al. (22) utilizing traditional multiple logistic regression to predict discharge timing in TKA reported an AUC of 0.773. Our CATBoost model’s discriminative ability (AUC = 0.832) compares favorably, underscoring the potential advantage of machine learning approaches in capturing complex, non-linear relationships for this clinical task.

Beyond performance metrics, a key advancement of our framework is its inherent interpretability. Decision curve analysis further affirmed CATBoost’s clinical utility, yielding higher net benefits across a wide risk threshold range (e.g., 0.1–0.8), while residual and calibration analyses supported its reliability for clinical deployment. Crucially, and unlike traditional regression models, our SHAP analysis provided model interpretability, identifying EF and preoperative ESR as the most influential features and confirming risk directionality consistent with clinical expectations. This provides clinicians not only with a predictive tool but also with actionable insights into individual patient risk factors for personalized care planning.

### 4.2 Predictors of delayed discharge following ambulatory total knee arthroplasty

In the context of ambulatory TKA, a lower EF, even within the normal range, is a significant risk factor for failing to achieve same-day or next-day discharge. It is critical to note that this association represents a gradient of risk and does not imply that most patients had an abnormal EF. A lower EF is a key predictor of underlying cardiac vulnerability, often necessitating further preoperative evaluation (23). This vulnerability is clinically manifested as a higher propensity for complications that preclude early discharge. Patients with conditions associated with impaired cardiac function, such as congestive heart failure (CHF), have been consistently shown to experience significantly longer hospital stays (24, 25) and a greater overall burden of postoperative morbidity, including cardiac and thromboembolic events (26). Specifically, the presence of CHF substantially increases the risk of major complications occurring beyond 24 h postoperatively, directly impacting the feasibility of short-stay protocols (27). Therefore, preoperative identification of a lower EF serves as a crucial indicator for enhanced perioperative risk stratification and patient counseling, highlighting the need for individualized care plans in ambulatory TKA settings. SHAP analysis reinforced this inverse relationship, showing that low EF values correlate with higher risk, and revealed nuanced interactions, e.g., elevated EF generally reduces risk, but when combined with high preoperative ESR, it may slightly increase risk probability, though the effect is minimal.

The association between prolonged LOS and a history of diabetes is well-established. Patients with diabetes undergoing surgery frequently experience insulin resistance and sustained hyperglycemia, which elevate the risk of postoperative complications and contribute to extended hospitalization (28, 29). Shohat et al. (30) further demonstrated that postoperative glycemic variability independently predicts longer hospital stays. In a large retrospective analysis of 210,075 same-day total knee arthroplasty procedures, Johnson et al. (31) identified diabetes mellitus as a significant predictor of failure to discharge within 24 hours. SHAP analysis substantiated this finding, indicating that diabetes substantially increases risk, with the effect amplified at higher EF values, underscoring its role as a key modifiable factor.

Preoperative renal dysfunction, indicated by a reduced estimated glomerular filtration rate (eGFR), significantly predicts failure to achieve same-day discharge after ambulatory TKA. In elderly patients, age-related decline in renal function impairs the clearance of anesthetic and perioperative medications. This pharmacokinetic alteration prolongs drug exposure, increasing the risk of adverse effects such as postoperative nausea and vomiting, sedation, or delirium, which may delay functional recovery and discharge readiness (32). Moreover, patients with chronic kidney disease (CKD), particularly those with an eGFR below 30 mL/min/1.73 m^2^, face substantially higher risks of systemic complications, including cardiovascular events, infection, and need for transfusion, all of which contribute to extended hospitalization (33–35). Even moderate reductions in eGFR (e.g., < 60 mL/min/1.73 m^2^) have been associated with prolonged LOS and increased morbidity after joint arthroplasty (33). Therefore, integrating preoperative eGFR assessment into patient selection protocols for ambulatory TKA is essential to identify high-risk individuals, optimize medication management, and reduce the likelihood of discharge delays. SHAP analysis confirmed that lower eGFR values increase risk, though its interaction with EF was negligible, supporting eGFR as an independent predictor.

An elevated preoperative ESR is significantly associated with prolonged LOS following TKA. This relationship stems from ESR’s role as a marker of systemic inflammation, where a high preoperative level indicates a greater baseline inflammatory burden, often linked to more severe joint disease and potentially slower postoperative recovery (36). The physiological response to TKA involves a sharp rise in ESR postoperatively, which resolves slowly, contributing to delayed functional milestones (37–39). Additionally, elevated ESR may raise clinical concern for complications like periprosthetic joint infection, necessitating extended observation (38, 40, 41). Recent evidence from an enhanced recovery after surgery (ERAS) model further confirms that a preoperative ESR > 15 mm/h independently predicts increased LOS, reinforcing its utility as a prognostic factor (42). SHAP analysis highlighted ESR’s critical role, characterized by a threshold effect, and showed that elevated ESR coupled with higher EF significantly decreases overall risk, emphasizing the importance of feature interactions in risk stratification.

The Barthel Index (BI), a widely used instrument for assessing functional independence, has been consistently demonstrated as a significant risk factor for prolonged LOS following TKA. In a retrospective analysis of 353 patients, a lower Modified BI score at admission was directly associated with an extended LOS in the female subgroup, emphasizing its predictive value for delayed discharge in this population (43). This relationship was further corroborated by a large-scale study involving 5,831 patients across multiple institutions, which identified the BI as a statistically significant influencer of LOS (*p* < 0.001) through multivariate regression analysis, indicating that poorer preoperative functional status correlates with longer hospitalization durations (43). Collectively, these findings underscore the utility of the BI in preoperative risk assessment to identify individuals susceptible to extended LOS, thereby aiding in the optimization of discharge planning and healthcare resource management. SHAP analysis validated the inverse association between BI and risk, with values exceeding 90 conferring a protective effect, and indicated limited interaction with EF, reinforcing BI’s standalone predictive value.

### 4.3 Clinical implications and distinction of predictors

A key implication of our findings is the distinction between modifiable and non-modifiable predictors, which directs distinct clinical actions. Among the factors identified, the Barthel Index and a history of diabetes represent potentially modifiable risk factors. The functional status captured by the Barthel Index may be improved through targeted prehabilitation programs prior to surgery. Similarly, glycemic control in patients with diabetes can be optimized perioperatively. These modifiable factors should be the primary focus for interventions aimed at reducing the risk of delayed discharge. In contrast, ejection fraction, preoperative eGFR, and elevated ESR are largely non-modifiable patient characteristics that serve as excellent tools for risk stratification. They are invaluable for preoperative identification of high-risk patients, allowing for enhanced counseling, optimized perioperative management (e.g., medication dosing in renal impairment), and efficient resource allocation within the ambulatory pathway, even if the factors themselves cannot be changed. This distinction enables clinicians to separate patients who may benefit from preoperative optimization from those for whom advanced care planning is the most appropriate strategy.

### 4.4 Limitations and cautious interpretation

Several limitations should be considered when interpreting our findings. First, while machine learning approaches such as SHAP can reveal robust associations between variables and outcomes, they do not establish causality. For instance, the relationship between diabetes and delayed discharge may reflect residual confounding from unmeasured variables (e.g., peripheral neuropathy or subclinical cardiovascular disease) rather than direct causation. Second, the exclusion of certain clinically relevant variables — such as glycated hemoglobin (HbA1c) due to high rates of missingness — may have limited the comprehensiveness of our metabolic risk profiling. Finally, the single-center design and moderate sample size (*n* = 449) may affect the generalizability of our model, as institutional-specific protocols and perioperative practices could influence discharge outcomes independently of patient-level factors.

### 4.5 Future research directions

Future work should prioritize multi-center validation cohorts (> 2,000 cases) to assess portability, followed by the development of a clinically deployable tool (e.g., a standalone web application or an integrated EHR plug-in) that calculates a real-time risk score using the five featured variables to alert clinicians at the point of care. Prospective trials integrating SHAP-based scores into clinical workflows, and causal mediation analyses to disentangle feature effects. Despite limitations, our integration of machine learning with functional biomarkers represents a step toward personalized discharge optimization in ambulatory TKA.

## 5 Conclusion

In summary, this study demonstrates that machine learning models, particularly CATBoost, can effectively predict delayed discharge following ambulatory total knee arthroplasty by leveraging five key preoperative variables: ejection fraction, diabetes status, estimated glomerular filtration rate, erythrocyte sedimentation rate, and Barthel Index. These factors collectively provide a robust framework for identifying high-risk patients, enabling targeted preoperative optimization and individualized discharge planning. SHAP analysis enhanced model interpretability by elucidating feature interactions, such as the modulating role of EF, which strengthen the clinical credibility of the model. While limitations such as single-center data and associative inferences require cautious interpretation, our findings underscore the potential of integrating predictive analytics into clinical practice to enhance resource allocation and patient outcomes in short-stay arthroplasty protocols. Future efforts should focus on external validation and prospective implementation to translate these insights into actionable care pathways.

## Data Availability

The raw data supporting the conclusions of this article will be made available by the authors, without undue reservation.

## Supplementary material

Supplementary Table 1 Comparison of baseline characteristics by discharge group and dataset.

Supplementary Table 2 Logistic_regression_results.

Supplementary Table 3 Final hyperparameter configurations for all models.
