# Supplementary material for: A SHAP-interpretable machine learning framework for predicting delayed discharge in ambulatory total knee arthroplasty: comparative validation of 14 models
Source: Front Med (Lausanne). 2025 Nov 5;12:1714792. doi: 10.3389/fmed.2025.1714792 (PMC12626822; doi:10.3389/fmed.2025.1714792)
Supplement: Supplementary Table 1 — Comparison of baseline characteristics by discharge group and dataset. [file Table_1.docx]

|  | **Ontime** | **Delayed** | **p** | **Validation Set** | **Training Set** | **p** |
| --- | --- | --- | --- | --- | --- | --- |
|  | ***N=279*** | ***N=170*** |  | ***N=134*** | ***N=315*** |  |
| EF | 63.0 [61.0;66.0] | 61.0 [59.0;63.0] | <0.001 | 62.0 [60.0;66.0] | 63.0 [60.0;65.0] | 0.848 |
| Gender: Female | 227 (81.4%) | 141 (82.9%) | 0.768 | 111 (82.8%) | 257 (81.6%) | 0.857 |
| Age | 64.0 [58.0;69.0] | 63.5 [58.0;69.0] | 0.424 | 64.0 [58.0;69.0] | 64.0 [58.0;69.0] | 0.679 |
| Disease Duration | 7.00 [4.00;10.0] | 9.00 [4.00;10.0] | 0.194 | 8.00 [4.00;10.0] | 8.00 [4.00;10.0] | 0.641 |
| Knee Flexion Angle: <90° | 60 (21.5%) | 53 (31.2%) | 0.029 | 33 (24.6%) | 80 (25.4%) | 0.958 |
| Knee Extension Deficit:>10° | 26 (9.32%) | 28 (16.5%) | 0.035 | 14 (10.4%) | 40 (12.7%) | 0.608 |
| Contralateral Arthroplasty History:Y | 52 (18.6%) | 27 (15.9%) | 0.538 | 24 (17.9%) | 55 (17.5%) | 1.000 |
| Preop-Hb | 136 [128;144] | 135 [126;144] | 0.235 | 139 [128;145] | 135 [127;144] | 0.139 |
| Preop-PLT | 225 [180;268] | 232 [198;272] | 0.054 | 232 [190;280] | 228 [184;268] | 0.225 |
| Preop-CRP | 2.29 [1.19;4.53] | 3.59 [1.44;9.38] | 0.001 | 2.42 [1.24;4.78] | 2.68 [1.25;5.79] | 0.413 |
| Preop-eGFR | 89.0 [76.0;96.0] | 85.0 [76.0;89.0] | 0.001 | 87.0 [77.0;95.0] | 87.0 [76.0;93.5] | 0.533 |
| Preop-Alb | 42.7 [40.9;44.3] | 42.6 [40.0;44.8] | 0.488 | 42.8 [41.2;44.6] | 42.6 [40.5;44.5] | 0.312 |
| Preop-ESR | 14.0 [8.00;22.5] | 17.0 [10.0;27.8] | 0.001 | 14.0 [7.00;23.0] | 16.0 [9.00;25.0] | 0.079 |
| ASA: 2 | 218 (78.1%) | 132 (77.6%) | 0.997 | 112 (83.6%) | 238 (75.6%) | 0.080 |
| ASA: 3 | 61 (21.9%) | 38 (22.4%) |  | 22 (16.4%) | 77 (24.4%) |  |
| Surgical Laterality: Right | 128 (45.9%) | 89 (52.4%) | 0.217 | 73 (54.5%) | 144 (45.7%) | 0.110 |
| Anesthesia Type: General anesthesia | 186 (66.7%) | 120 (70.6%) | 0.500 | 86 (64.2%) | 220 (69.8%) | 0.294 |
| Anesthesia Type: Local anesthesia | 17 (6.09%) | 12 (7.06%) |  | 12 (8.96%) | 17 (5.40%) |  |
| Anesthesia Type: Combined anesthesia | 76 (27.2%) | 38 (22.4%) |  | 36 (26.9%) | 78 (24.8%) |  |
| Occupation:Unemployed | 181 (64.9%) | 111 (65.3%) | 0.729 | 85 (63.4%) | 207 (65.7%) | 0.692 |
| Occupation:Manual labor job | 60 (21.5%) | 39 (22.9%) |  | 34 (25.4%) | 65 (20.6%) |  |
| Occupation: Intellectual labor job | 8 (2.87%) | 2 (1.18%) |  | 2 (1.49%) | 8 (2.54%) |  |
| Occupation: Retired | 30 (10.8%) | 18 (10.6%) |  | 13 (9.70%) | 35 (11.1%) |  |
| BMI: <18.5 | 3 (1.08%) | 3 (1.76%) | 0.847 | 3 (2.24%) | 3 (0.95%) | 0.224 |
| BMI: ≥18.5 | 96 (34.4%) | 57 (33.5%) |  | 52 (38.8%) | 101 (32.1%) |  |
| BMI: ≥24 | 119 (42.7%) | 69 (40.6%) |  | 48 (35.8%) | 140 (44.4%) |  |
| BMI: ≥28 | 61 (21.9%) | 41 (24.1%) |  | 31 (23.1%) | 71 (22.5%) |  |
| Educational Attainment: Illiterate | 102 (36.6%) | 63 (37.1%) | 0.983 | 55 (41.0%) | 110 (34.9%) | 0.738 |
| Educational Attainment: Primary school | 122 (43.7%) | 72 (42.4%) |  | 56 (41.8%) | 138 (43.8%) |  |
| Educational Attainment: Junior high school | 41 (14.7%) | 27 (15.9%) |  | 18 (13.4%) | 50 (15.9%) |  |
| Educational Attainment: Senior high school | 9 (3.23%) | 6 (3.53%) |  | 3 (2.24%) | 12 (3.81%) |  |
| Educational Attainment: College | 5 (1.79%) | 2 (1.18%) |  | 2 (1.49%) | 5 (1.59%) |  |
| Body Temperature | 36.3 [36.2;36.5] | 36.3 [36.2;36.5] | 0.594 | 36.3 [36.2;36.5] | 36.3 [36.2;36.5] | 0.979 |
| Pulse Rate | 78.0 [72.0;87.0] | 81.5 [74.0;89.8] | 0.030 | 81.3 (11.8) | 79.9 (10.6) | 0.224 |
| Systolic Blood Pressure | 131 (15.2) | 133 (17.3) | 0.141 | 132 (15.4) | 131 (16.3) | 0.467 |
| Diastolic Blood Pressure | 79.8 (9.68) | 81.2 (10.8) | 0.172 | 81.8 (9.64) | 79.6 (10.3) | 0.034 |
| Smoking Status:Y | 35 (12.5%) | 14 (8.24%) | 0.206 | 12 (8.96%) | 37 (11.7%) | 0.482 |
| Alcohol Use:Y | 26 (9.32%) | 10 (5.88%) | 0.262 | 12 (8.96%) | 24 (7.62%) | 0.774 |
| Hypertension:Y | 110 (39.4%) | 78 (45.9%) | 0.213 | 52 (38.8%) | 136 (43.2%) | 0.451 |
| Diabetes Mellitus: Y | 20 (7.17%) | 32 (18.8%) | <0.001 | 19 (14.2%) | 33 (10.5%) | 0.337 |
| Osteoporosis:Y | 87 (31.2%) | 47 (27.6%) | 0.492 | 43 (32.1%) | 91 (28.9%) | 0.572 |
| Emphysema:Y | 7 (2.51%) | 6 (3.53%) | 0.569 | 4 (2.99%) | 9 (2.86%) | 1.000 |
| Pneumonia:Y | 21 (7.53%) | 7 (4.12%) | 0.212 | 5 (3.73%) | 23 (7.30%) | 0.223 |
| History of Cerebral Infarction:Y | 9 (3.23%) | 5 (2.94%) | 1.000 | 4 (2.99%) | 10 (3.17%) | 1.000 |
| Coronary Artery Disease:Y | 35 (12.5%) | 18 (10.6%) | 0.637 | 11 (8.21%) | 42 (13.3%) | 0.168 |
| Knee Deformity:Y | 11 (3.94%) | 14 (8.24%) | 0.087 | 12 (8.96%) | 13 (4.13%) | 0.069 |
| Resting VAS Score | 2.00 [2.00;3.00] | 2.00 [2.00;3.00] | 0.895 | 2.00 [2.00;3.00] | 2.00 [2.00;3.00] | 0.625 |
| Barthel Index | 90.0 [85.0;95.0] | 90.0 [80.0;95.0] | 0.012 | 90.0 [85.0;95.0] | 90.0 [80.0;95.0] | 0.657 |
| Mobility Aid Requirement: Y | 28 (10.0%) | 26 (15.3%) | 0.131 | 20 (14.9%) | 34 (10.8%) | 0.283 |
| Data Source: Validation Set | 83 (29.7%) | 51 (30.0%) | 1.000 |  |  |  |
| Training Set | 196 (70.3%) | 119 (70.0%) |  |  |  |  |
| Discharge cohorts:Ontime |  |  |  | 83 (61.9%) | 196 (62.2%) | 1.000 |
| Delayed |  |  |  | 51 (38.1%) | 119 (37.8%) |  |
