# Supplementary material for: A SHAP-interpretable machine learning framework for predicting delayed discharge in ambulatory total knee arthroplasty: comparative validation of 14 models
Source: Front Med (Lausanne). 2025 Nov 5;12:1714792. doi: 10.3389/fmed.2025.1714792 (PMC12626822; doi:10.3389/fmed.2025.1714792)
Supplement: Supplementary Table 2 — Logistic_regression_results. [file Table_2.docx]

|  | Ontime (N=196) | Delayed (N=119) | OR  (univariable) | OR (multivariable) | OR  (final) |
| --- | --- | --- | --- | --- | --- |
| EF | 64.2 ± 4.5 | 61.0 ± 3.4 | 0.80 (0.74-0.86, p<.001) | 0.80 (0.74-0.87, p<.001) | 0.80 (0.74-0.87, p<.001) |
| Preop-eGFR | 85.0 ± 15.8 | 79.7 ± 16.0 | 0.98 (0.97-0.99, p=.006) | 0.98 (0.97-1.00, p=.068) | 0.98 (0.97-1.00, p=.068) |
| Preop-ESR | 18.2 ± 15.9 | 22.9 ± 17.1 | 1.02 (1.00-1.03, p=.016) | 1.02 (1.00-1.04, p=.024) | 1.02 (1.00-1.04, p=.024) |
| Diabetes Mellitus | 12 (6.1%) | 21 (17.6%) | 3.29 (1.55-6.96, p=.002) | 2.81 (1.23-6.40, p=.014) | 2.81 (1.23-6.40, p=.014) |
| Barthel Index | 88.9 ± 10.0 | 85.1 ± 12.4 | 0.97 (0.95-0.99, p=.004) | 0.98 (0.96-1.00, p=.101) | 0.98 (0.96-1.00, p=.101) |
