# Supplementary material for: A SHAP-interpretable machine learning framework for predicting delayed discharge in ambulatory total knee arthroplasty: comparative validation of 14 models
Source: Front Med (Lausanne). 2025 Nov 5;12:1714792. doi: 10.3389/fmed.2025.1714792 (PMC12626822; doi:10.3389/fmed.2025.1714792)
Supplement: Supplementary Table 3 — Final hyperparameter configurations for all models. [file Table_3.docx]

| Model | Parameter | Value |
| --- | --- | --- |
| RandomForest | mtry | 10 |
| GradientBoosting | nrounds | 100 |
| GradientBoosting | max_depth | 3 |
| GradientBoosting | eta | 0.1 |
| GradientBoosting | gamma | 0 |
| GradientBoosting | colsample_bytree | 0.7 |
| GradientBoosting | min_child_weight | 1 |
| GradientBoosting | subsample | 0.7 |
| SVM_Kernel | sigma | 0.1 |
| SVM_Kernel | C | 2 |
| LogisticModel | parameter | none |
| NeighborMethod | k | 9 |
| PLSModel | ncomp | 4 |
| BoostingMethod | n.trees | 50 |
| BoostingMethod | interaction.depth | 5 |
| BoostingMethod | shrinkage | 0.1 |
| BoostingMethod | n.minobsinnode | 5 |
| NeuralNet | size | 5 |
| NeuralNet | decay | 0.01 |
| BayesMethod | fL | 0 |
| BayesMethod | usekernel | TRUE |
| BayesMethod | adjust | 1 |
| DiscriminantModel | parameter | none |
| Lasso | alpha | 0 |
| Lasso | lambda | 0.1 |
| AdaptiveBoosting | mfinal | 50 |
| AdaptiveBoosting | maxdepth | 5 |
| AdaptiveBoosting | coeflearn | Breiman |
| XGBoost | nrounds | 50 |
| XGBoost | lambda | 1 |
| XGBoost | alpha | 1 |
| XGBoost | eta | 0.01 |
| CATBoost | depth | 6 |
| CATBoost | learning_rate | 0.05 |
| CATBoost | l2_leaf_reg | 5 |
| CATBoost | iterations | 105 |
